# Fluid-limiting treatment strategies among sepsis patients in the ICU: a retrospective causal analysis

**DOI:** 10.1186/s13054-020-2767-0

**Published:** 2020-02-22

**Authors:** Zach Shahn, Nathan I. Shapiro, Patrick D. Tyler, Daniel Talmor, Li-wei H. Lehman

**Affiliations:** 1 IBM Research, Yorktown Heights, NY USA; 2 MIT-IBM Watson AI Lab, Cambridge, USA; 3 Department of Emergency Medicine, Beth Israel Deaconess Medical Center, Boston, MA USA; 4 Department of Anesthesia, Critical Care and Pain Medicine, Beth Israel Deaconess Medical Center and Harvard Medical School, Boston, MA USA; 5 Institute for Medical Engineering and Science, Massachusetts Institute of Technology, Cambridge, MA USA

**Keywords:** Sepsis, Intravenous fluids, Resuscitation, Causal inference, Intensive care medicine, Emergency medicine

## Abstract

**Objective:**

In septic patients, multiple retrospective studies show an association between large volumes of fluids administered in the first 24 h and mortality, suggesting a benefit to fluid restrictive strategies. However, these studies do not directly estimate the causal effects of fluid-restrictive strategies, nor do their analyses properly adjust for time-varying confounding by indication. In this study, we used causal inference techniques to estimate mortality outcomes that would result from imposing a range of arbitrary limits (“caps”) on fluid volume administration during the first 24 h of intensive care unit (ICU) care.

**Design:**

Retrospective cohort study

**Setting:**

ICUs at the Beth Israel Deaconess Medical Center, 2008–2012

**Patients:**

One thousand six hundred thirty-nine septic patients (defined by Sepsis-3 criteria) 18 years and older, admitted to the ICU from the emergency department (ED), who received less than 4 L fluids administered prior to ICU admission

**Measurements and main results:**

Data were obtained from the Medical Information Mart for Intensive Care III (MIMIC-III). We employed a dynamic Marginal Structural Model fit by inverse probability of treatment weighting to obtain confounding adjusted estimates of mortality rates that would have been observed had fluid resuscitation volume caps between 4 L–12 L been imposed on the population. The 30-day mortality in our cohort was 17%. We estimated that caps between 6 and 10 L on 24 h fluid volume would have reduced 30-day mortality by − 0.6 to − 1.0%, with the greatest reduction at 8 L (− 1.0% mortality, 95% CI [− 1.6%, − 0.3%]).

**Conclusions:**

We found that 30-day mortality would have likely decreased relative to observed mortality under current practice if these patients had been subject to “caps” on the total volume of fluid administered between 6 and 10 L, with the greatest reduction in mortality rate at 8 L.

## Introduction

Sepsis is a commonly encountered problem in the emergency department (ED) and intensive care unit (ICU), inflicting substantial morbidity and mortality [1, 2]. One critical element of treating sepsis involves correcting hypovolemia and perfusion abnormalities using intravenous fluids and vasopressors. However, the optimal dosing and timing of fluid resuscitation in patients with sepsis remains unknown.

There is ongoing clinical debate and research in progress regarding whether to pursue a more liberal or restrictive fluid administration strategy [3]. The landmark study of early goal-directed therapy (EGDT) by Rivers et al. led to an era of liberal fluid administration [3], particularly as follow-on studies showed improved sepsis survival in hospitals that provided bundled sepsis care based on EGDT [4–6]. However, a growing body of observational literature [7–13] and several randomized trials (two in the developing world, one unblinded pilot trial in Europe) [14–16] evaluating the relationship between fluid administration and mortality suggest that large-volume fluid administration might be deleterious. These results are unable to guide current clinical management, as there is not a convincing control for confounding by indication in the observational studies, and several obstacles prevent generalization of the randomized trial results to sepsis patients in the developed world [17].

To better understand the effect of different fluid resuscitation strategies on patient outcomes, a multicenter, phase III, randomized trial (the CLOVERS study) is currently in progress; the results of this trial will not be available for several years [17, 18]. We believe that a causal analysis of a large observational dataset could help to inform the debate around fluid resuscitation decisions in the meantime, as well as inform our interpretation of the findings from the CLOVERS trial when they arrive. To that end, we performed a retrospective cohort study of ICU patients with sepsis at a tertiary center and used causal inference techniques to obtain confounding adjusted estimates of mortality outcomes that would result from imposing different limits on fluid volume administration (“caps”) during the first 24 h of ICU care. We hypothesized that certain caps on fluid resuscitation would cause decreases in 30-day mortality (compared to current practice) for our patient population.

## Methods

### Sample selection

Data were obtained from the Medical Information Mart for Intensive Care III (MIMIC-III) [19]. The database contains records from 38,597 distinct adult patients admitted to ICUs at the Beth Israel Deaconess Medical Center from 2001 to 2012. The database contains detailed information about vital signs, medication administration, ventilator settings, and other granular ICU-level data not typically available in retrospective data sets.

This study included MIMIC-III patients aged > 18 years with sepsis admitted to the ICU from the ED between 2008 and 2012 (the years when pre-admission ICU IV fluids were documented). We selected for sepsis patients using the definition from the Third International Consensus Definitions for Sepsis and Septic Shock (Sepsis-3), which includes suspected infection (defined by having both blood cultures drawn and antibiotics administered) and a Sequential Organ Failure Assessment (SOFA) score ≥ 2 [20]. Data extraction adhered to the original Sepsis-3 protocol [20] and a prior study in identifying the Sepsis-3 cohort in MIMIC-III [21]. Patients suspected of infection more than 24 h after ICU admission were excluded, as were patients with missing antibiotics and blood culture samples [21]. Patients with secondary (or greater) admissions were excluded to avoid repeated measures.

We excluded patients who had already received greater than 4 L of IV fluids prior to ICU admission, as these patients would have already violated some of our treatment strategies of interest at baseline. We excluded patients documented as receiving 0 L of fluid in the ED, as this likely indicated failure to record. As mentioned above, only ED admissions were included; patients transferred from another hospital to the ICU or admitted to the ICU from the operating room or hospital ward were excluded. Out-of-hospital mortality dates in MIMIC-III were obtained from the linked Social Security Administration Death Master File.

We extracted the following variables from the MIMIC database for all patients: baseline demographic information (age, gender, race), ICU details (continuous vital sign monitoring, fluid inputs and outputs, fluids and medications administered, laboratory values, and respiratory support), all additional variables needed to calculate the SOFA score, and components of the Elixhauser comorbidity index.

### Overall analysis strategy: emulating a randomized clinical trial

This was a retrospective causal cohort study of ICU patients with sepsis at a large tertiary center. The goal of causal inference generally is to emulate a hypothetical (and not necessarily practical) randomized controlled trial (RCT) using observational data [22]. The hypothetical RCT we sought to emulate in this study has many treatment arms. In one treatment arm of the RCT, physicians would be instructed to deliver “usual care” or “current practice,” i.e., make no modification to the treatment decisions they would make when they are not participating in a RCT. Care followed the Surviving Sepsis Campaign [23] guidelines at the time. However, because these guidelines are not strict concerning fluid administration, there was a good deal of practice variability. Each other treatment arm would correspond to a different cut-off or cap on total fluid volume received by the end of the first 24 h after ICU admission. The caps range from 4 L to 12 L. Patients randomly assigned to a treatment arm would be treated according to usual care until they approached the arm’s fluid volume cap, at which point they would be prevented from receiving any further fluids. (If a patient assigned to a fluid cap of 5 L, for example, would not exceed 5 L of fluids under usual care, then their treatment would not be altered by participation in the RCT.) “Baseline” for our hypothetical RCT is ICU admission, and the inclusion and exclusion criteria are described in the “Sample selection” section. Our study estimates the 30-day mortality that would be observed in each arm of this hypothetical RCT.

The unadjusted observed mortality rate in the cohort is an unbiased estimate of the mortality rate that would be observed in the usual care arm of our ideal RCT. The primary challenge of estimating the mortality rates in the other *counterfactual* RCT arms from observational data is confounding—that is, the tendency for patients to receive differential amounts of fluids because they had different clinical characteristics or comorbidities that were associated with the outcome. Our objective was therefore to obtain confounding-adjusted estimates of the mortality rates that would have been observed had fluid volume cutoffs between 4 L and 12L been imposed on the population.

### Confounding-adjusted estimate of mortality rates

We fit a dynamic Marginal Structural Model (dyn-MSM) to estimate our causal quantities of interest [24, 25]. We adjusted for confounding, i.e., accounted for the fact that patients who follow different treatment strategies tend to have different characteristics, by inverse probability of treatment weighting [25–27]. Here, we give a brief intuitive description of the method, which we describe in more technical detail in Additional file 1. Estimation of causal effects and quantification of uncertainty proceed in three steps.

- Step 1: *Separately estimate mortality rate under each cap*. To estimate the counterfactual mortality rate under a particular fluid resuscitation cap, we take the weighted average of mortality among patients whose fluid volumes were actually under the cap, weighting each patient appropriately so that they represent not only themselves but also all similar patients who exceeded the cap. Appropriate weighting requires a predictive model for the probability of remaining below the cap at each time given patient history of confounders up to that time. Each patient’s weight is then the inverse of the product of these probabilities over all time steps. We used a boosted trees model to generate the probabilities [28].
- Step 2: *Smooth the separate counterfactual mortality rate estimates*. Having obtained separate estimates of counterfactual population mortality rates corresponding to a range of fluid volume caps, we next incorporate the assumption that mortality rate varies smoothly as a function of fluid volume cap. This allows us to “borrow strength” across estimates of mortality under different caps and improve the precision of our estimates for all caps. We specify that counterfactual mortality rate as a function of volume cap is described by a spline regression function. Details of estimation of the coefficients of the spline are left to Additional file 1. Given the spline coefficient estimates, we estimate counterfactual mortality under any volume cap by simply plugging the volume cap value into the spline function.
- Step 3: *Quantifying uncertainty*. We repeat steps 1 and 2 on 500 bootstrap samples of the data to obtain confidence intervals for the estimated effect of each cap. We obtain a simultaneous confidence interval over all caps in the range using the method from Appendix C of [29].

For our results to have a causal interpretation, it is important that the covariate history input to the predictive model in step 1 contains all variables that are (1) important drivers of fluid treatment decisions and (2) associated with mortality. Since essentially every variable in our dataset is associated with mortality, our focus in covariate selection was to include all drivers of treatment. We included both baseline and time-varying variables. Baseline variables were age, gender, ethnicity, weight, body mass index, service unit, Elixhauser comorbidities, and fluid volume administered prior to ICU admission. Time-varying variables included vital signs, lab values (platelets, creatinine, lactate), fluid volume administered in the previous hour, total fluid volume through the previous hour, urine output, Glasgow Coma Scale (GCS; both combined score and individual components), SOFA score (combined score and individual components), estimated 30-day mortality (based on a boosted trees predictive model fit to pre-treatment variables), and respiratory interventions (oxygen therapy, non-invasive and invasive mechanical ventilation). For each time-varying variable, we adjusted for its most recently measured value, time since it was last measured, its value the previous hour, and its running mean, maximum, and minimum. For a full list of variables that we adjusted for, see Appendix B in Additional file 1.

Our constructed dataset contained a row for each hour after admission for each patient. At each hour, the most recent measurement of each variable was recorded, as this is the value that the doctor is aware of and might influence treatment decisions. For each variable, time points prior to any measurements were entered as “NA” to indicate “not available,” also reflecting the doctor’s knowledge about those variables at the time. Boosted trees accept NAs as inputs and estimate probabilities conditional on missingness. Covariate measurements made in the same hour as but following a treatment action (i.e., a shift in fluid resuscitation rate) were shifted to the following hour so as not to adjust for post-treatment variables.

As a sensitivity analysis, we repeated our analysis under alternative modeling decisions. We imputed all missing covariate values through multiple imputation and applied logistic regression to estimate treatment probabilities when computing inverse probability of treatment weights. The details of this approach are described in Appendix D in Additional file 1.

## Results

**Fig. 1:**
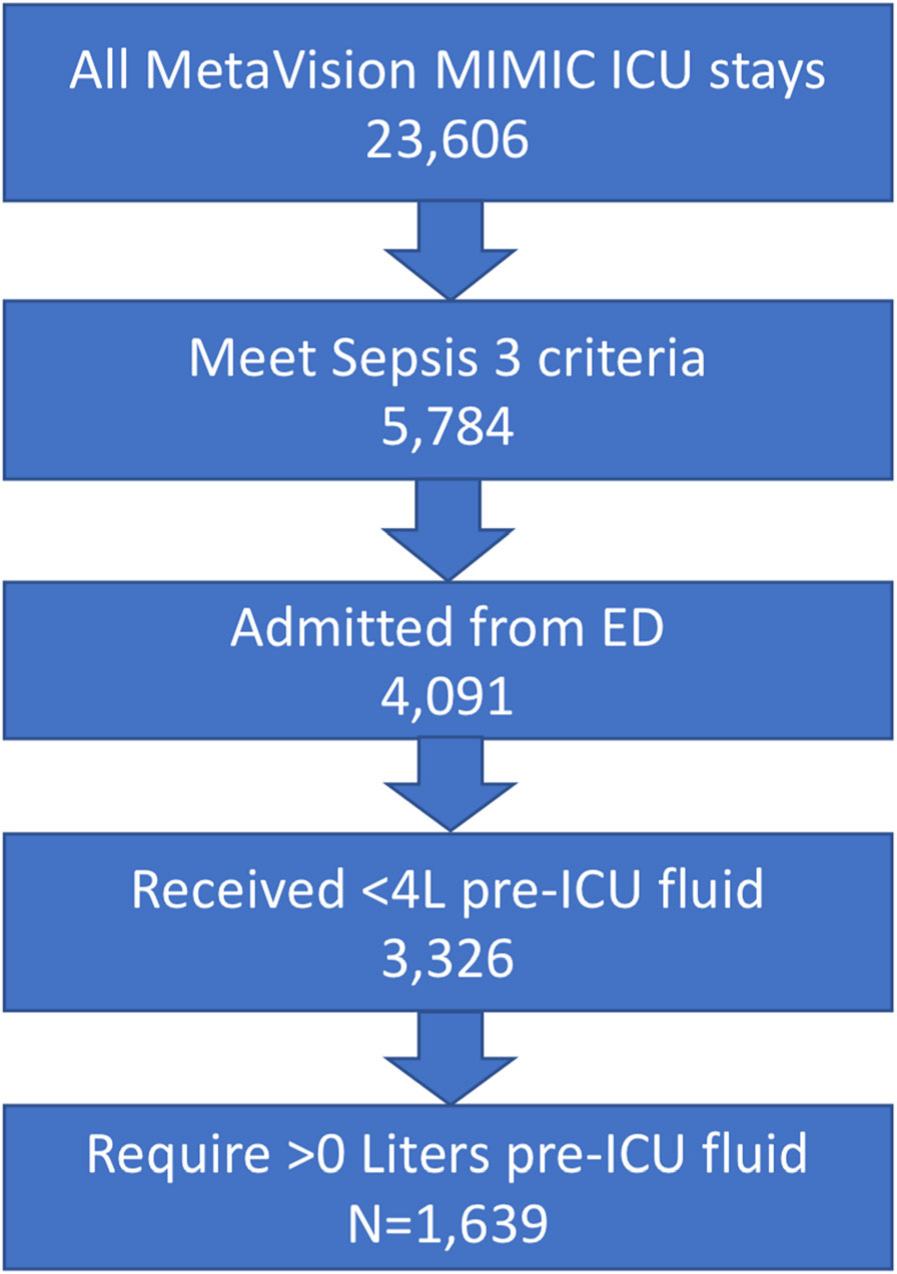
Cohort construction flow chart

**Fig. 2:**
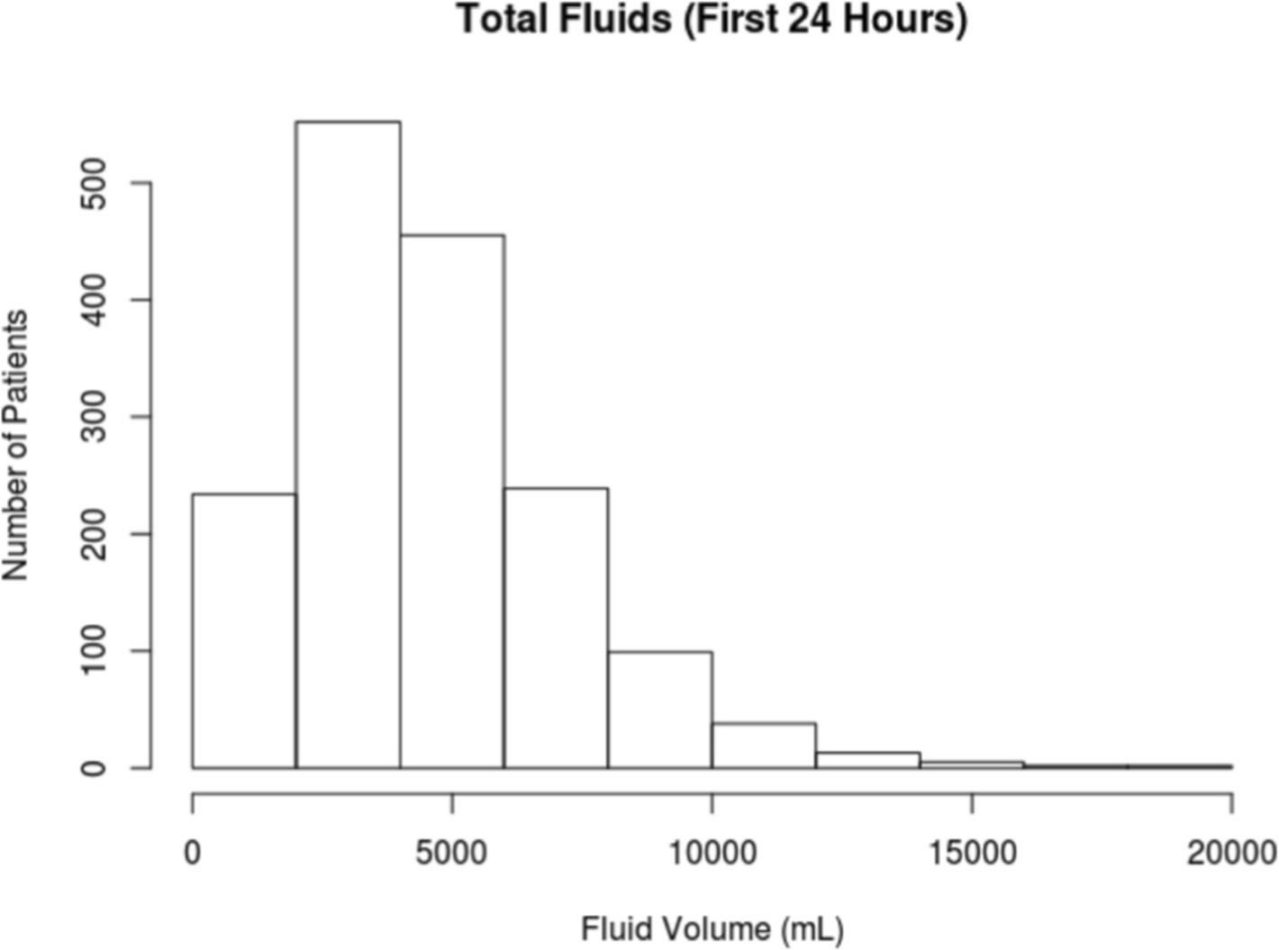
Distribution of fluid volumes received in the first 24 h. Distribution of total fluid volume administered by 24 h after ICU admission in our cohort

The MIMIC-III database contained 5784 adult patients meeting Sepsis-3 criteria upon ICU admission between 2008 and 2012 [21]. Among these sepsis patients, 4091 patients were admitted to the ICU from the ED. There were 765 patients who were excluded for receiving more than 4 L of IV fluid prior to ICU admission, and 1687 patients were recorded as receiving 0 L pre-ICU fluid and were also excluded. The remaining 1639 patients comprising our cohort (see Fig. 1) received a median of 3.5 L IVF (interquartile range, 1.6–6.7); the distribution of fluid volumes is shown in Fig. 2. Patients received a broad range of IV fluids during the first 24 h, primarily traditional crystalloid solutions (0.9% sodium chloride, lactated Ringer’s, or variations). Characteristics of the patient population broken down by fluid volume are shown in Table 1. Observed mortality in our cohort was 17%.

**Table 1:** Cohort summary

Characteristics	Mean (sd) among patients with total fluids < 4 L (*N* = 786)	Mean (sd) among patients with total fluids < 6 L (*N* = 1241)	Mean (sd) among patients with total fluids < 8 L (*N* = 1480)	Mean (sd) among patients with total fluids < 10 L (*N* = 1579)	Mean (sd) among all patients (*N* = 1639)
Proportion male	0.50	0.54	0.54	0.54	0.54
Mean (sd) MAP (mmHg)	83 (16)	84 (17)	83 (18)	83 (19)	83 (19)
Mean (sd) weight (Kgs)	80 (31)	81 (29)	81 (28)	81 (28)	81 (28)
Mean (sd) age (years)	69 (18)	68 (18)	67 (18)	67 (18)	67 (18)
Mean (sd) SOFA	6.4 (2.8)	6.7 (3.0)	7.0 (3.1)	7.1 (3.2)	7.3 (3.3)
Mean (sd) Cardiovascular SOFA subscore	1.1 (0.8)	1.2 (1.0)	1.3 (1.1)	1.3 (1.1)	1.4 (1.2)
Mean (sd) renal SOFA subscore	2.5 (1.5)	2.4 (1.6)	2.4 (1.5)	2.5 (1.5)	2.5 (1.5)
Mean (sd) CNS SOFA subscore	1.6 (1.4)	1.8 (1.4)	1.8 (1.5)	1.8 (1.5)	1.9 (1.5)
Mean (sd) respiration SOFA subscore	0.5 (1.1)	0.6 (1.1)	0.6 (1.1)	0.6 (1.1)	0.7 (1.2)
Mean (sd) coagulation SOFA subscore	0.4 (0.8)	0.5 (0.8)	0.5 (0.8)	0.5 (0.8)	0.5 (0.8)
Mean (sd) liver SOFA subscore	0.3 (0.7)	0.3 (0.8)	0.4 (0.8)	0.4 (0.8)	0.4 (0.8)
Proportion Caucasian	0.74 (0.4)	0.73 (0.4)	0.73 (0.4)	0.73 (0.4)	0.73 (0.4)
Mean (sd) Elixhauser	2.4 (1.5)	2.4 (1.5)	2.4 (1.5)	2.4 (1.5)	2.4 (1.5)
Mean (sd) GCS (baseline)	12.9 (3.4)	12.4 (3.8)	12.2 (4.0)	12.2 (4.0)	12.2 (4.1)
Mean (sd) pre-ICU fluid volume (L)	1.4 (0.8)	1.8 (1.0)	2.0 (1.1)	2.1 (1.1)	2.1 (1.1)
Proportion with metastatic cancer	0.06	0.06	0.07	0.07	0.06
Proportion with diabetes	0.06	0.06	0.06	0.06	0.06
Proportion starting vasopressors in first 24 h	0.12	0.18	0.22	0.24	0.26
Proportion initiating MV in first 24 h	0.30	0.35	0.36	0.37	0.38

Characteristics of patients in the cohort broken down by cap on total fluids received by the end of the first 24 h in the ICU. SOFA subscores are computed as maxima over the first 24 h, and SOFA is computed as their sum

**Fig. 3:**
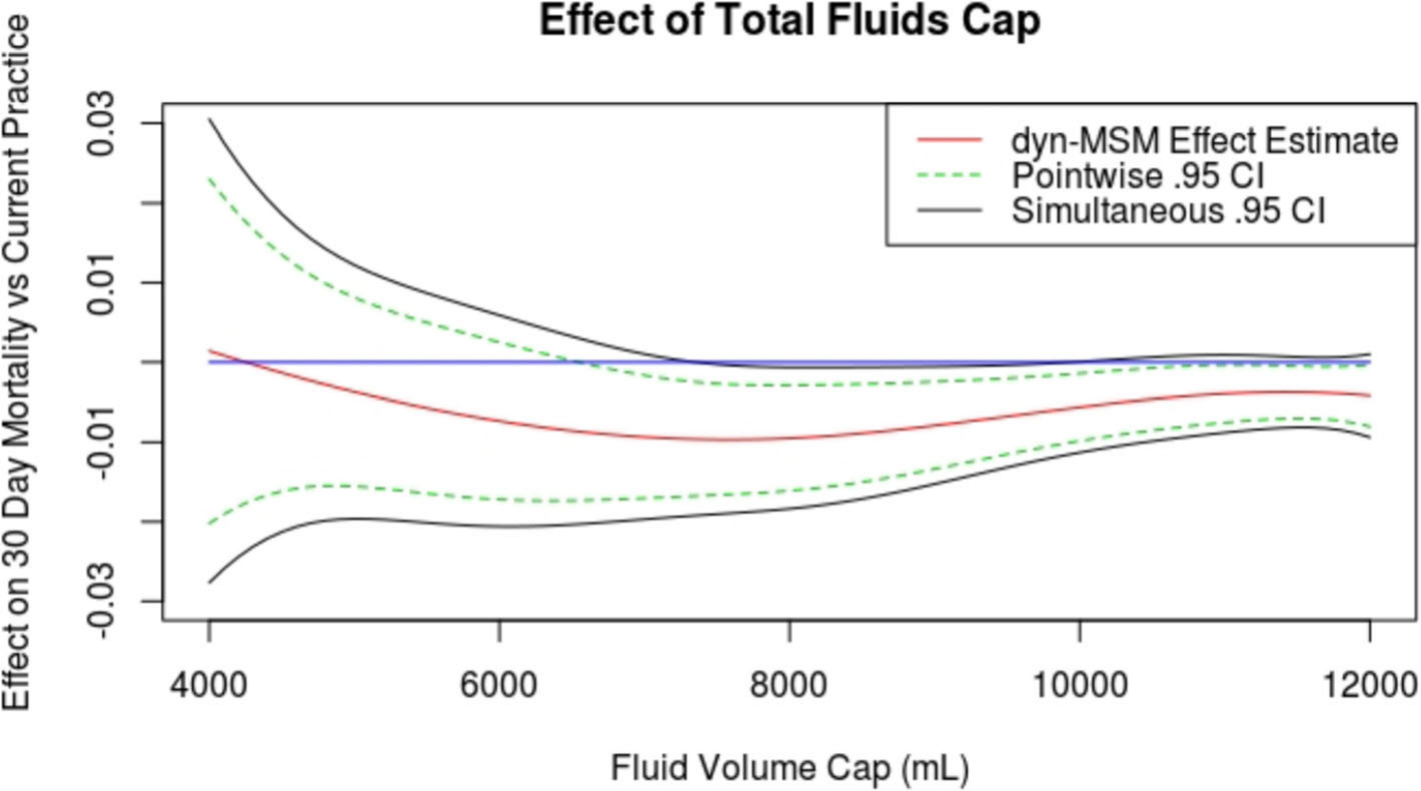
Effects of total fluids caps. Estimated effect on 30-day mortality compared to current practice (*Y*-axis) had total fluids through the first 24 h after ICU admission been capped at each volume (*X*-axis) between 4 L and 12 L. Blue line indicates 0

We also report estimated mortality under a range of fluid volume caps. For each volume between 4 L and 12 L (*X*-axis), Fig. 3 shows the estimated effect on 30-day mortality compared to current practice (*Y*-axis) had total fluids through the first 24 h after ICU admission been capped at that volume. Negative values on the *Y*-axis correspond to reductions in mortality. Caps between about 6 L and 10 L are estimated to reduce mortality rate by approximately 1%, with the 30-day mortality rate under current practice approximately 17%. Expected effects on mortality for selected fluid caps are shown in Table 2. Volume caps near 8 L are estimated to lower mortality rates most (Table 2). Harmful effects are least compatible with the data for caps between 8 L and 10 L. However, the data are consistent with negligible beneficial effect sizes even in this range.

**Table 2:** Selected treatment effect estimates

Fluid volume cap	Estimated effect on 30-day mortality compared to current practice (.95 CI)
4 L	0.1% (− 2.0%, 2.3%)
5 L	− 0.4% (− 1.6%, 0.8%)
6 L	− 0.7% (− 1.7%, 0.2%)
7 L	− 0.9% (− 1.7%, − 0.2%)
8 L	− 1.0% (− 1.6%, − 0.3%)
9 L	− 0.8% (− 1.3%, − 0.2%)
10 L	− 0.6% (− 1.0%, − 0.1%)
11 L	− 0.4% (− 0.8%, 0.0%)
12 L	− 0.4% (− 0.8%, 0.0%)

Estimated effects on 30-day mortality of selected fluid volume caps compared to current practice

Beyond 12 L, caps are estimated to have little to no effect compared to current practice, which is natural given the fact that few patients received that much fluid under current practice (see the histogram in Fig. 1), and hence few patients would have their treatment altered by such high-volume caps. Effects of caps at lower volumes have higher associated uncertainties because relatively few sick patients were actually treated in accordance with these caps, making it more uncertain what would happen if they had been.

Table 3 in Appendix B in Additional file 1 reports the relative feature importance of the most important covariates in our boosted trees treatment prediction model used for confounding adjustment by inverse probability weighting [30, 31]. Variables assigned high importance were useful for predicting treatment and were likely well adjusted for as confounders. To the extent that variables thought to be important confounders appear in this table, it is a reassuring indication that we appropriately adjusted for confounding bias by observed variables.

Results of a sensitivity analysis employing multiple imputation for missing data and logistic regression for our treatment probability model can be found in Appendix D of Additional file 1. They were very similar to those presented in the main body of the paper.

## Discussion

In this causal cohort study in a large critical care database, we found that 30-day mortality would have likely decreased relative to observed mortality under current practice if the patients in our cohort were subjected to “caps” on the total volume of fluid administered by the end of the first 24 h in the ICU. Specifically, we found that caps between 6 and 10 L would lead to the most pronounced reductions in 30-day mortality, with the greatest reduction at 8 L (− 1.0% mortality, 95% CI − 1.6 to − 0.3%). An important strength of this study compared to past work was the use of causal inference methods to rigorously adjust for time-varying confounding in observational data.

As we hypothesized, we found a beneficial effect of fluid resuscitation caps on 30-day mortality. We believe this is due to the deleterious effects of excessive fluid resuscitation in septic patients and that our study adds to mounting evidence that large positive fluid balances are harmful. Potential mechanisms of this harm include soft tissue and organ edema, worsened by endovascular leak; this leads to respiratory, cardiac, and renal failure [32–34]. Additionally, crystalloid resuscitation may directly injure the glycocalyx, which could contribute to organ failures [35].

Our findings add additional evidence to retrospective studies suggesting that large positive fluid balances may be deleterious [7–13]. Several authors retrospectively address the association between exposure to positive fluid balance at 24 h and the outcome of mortality. In a retrospective analysis of fluid resuscitation in 325 patients with septic shock, Micek et al. found that patients in the highest quartile of positive fluid balance at 24 h after shock recognition had increased in-hospital mortality compared with those in the first and second quartile [8]. Sadaka et al. retrospectively studied 350 adults with septic shock and found that patients with 24-h fluid positive fluid balances of 6–12, 12–18, and 18–24 L had increased mortality relative to patients with a balance of less than 6 L [9]. de Oliveira et al. retrospectively examined the fluid balance between 24 and 48 h after first recognition of *organ dysfunction* in septic patients in the ICU and found that fluid balance > 3 L was associated with increased hospital mortality [11].

Several other (also associational) analyses consider slightly different exposures or outcomes than our study. Boyd et al. retrospectively examined 12-h fluid balance (ICU patients with septic shock, *n* = 778) and found that those in the lower quartiles of fluid balance had lower mortality [7]. Acheampong and Vincent retrospectively examined the exposure of fluid balance in the first 7 days (ICU patients with sepsis and at least one organ failure, *n* = 173) and found an association between increasing fluid balance and mortality [10]. Kelm et al. retrospectively evaluated for signs of fluid overload on exam on hospital day 1 (ICU patients with sepsis and at least one organ failure, *n* = 405) and found that at least one sign of fluid overload was associated with increased in-hospital mortality [12]. Finally, Sakr et al. prospectively examined the association between net fluid balance at 24 and 72 h (ICU patients with sepsis and at least one organ failure, *n* = 1808), finding that higher fluid balance at 72 but not 24 h was associated with increased 28-day mortality [13].

Our study builds on this literature by using causal inference techniques applied to rich longitudinal data to explicitly estimate causal effects of fluid limiting treatment strategies. All observational studies are vulnerable to confounding by indication. Our study was less susceptible to this bias than past observational studies on fluid administration for two reasons. First, the MIMIC dataset we used in our analysis contained granular temporal detail on a large number of clinical variables, which allowed us to adjust for more confounding variables than past studies. Second, exploiting the temporal detail of the MIMIC data, we employed causal inference methods that appropriately handled the time-varying nature of the problem. Even where prior retrospective studies attempt to control for patient-level variables using logistic regression [11, 12] or proportional hazard models [7–10], these methods only control for baseline confounders, not confounders that evolve as the illness course progresses in the ICU. For example, patients A and B with septic shock may have similar baseline characteristics, but at hour 12, patient A may have improved, while patient B may have worsened. This change in clinical condition affects the propensity of these patients to receive further fluids between hours 12 and 24 and is also clearly associated with mortality. By using the methods outlined above, we have accounted for such time-varying confounding. The fact that our study produces findings consistent with prior retrospective studies regarding fluid balance should encourage further interest in evaluating different fluid resuscitation strategies in RCTs. Pending evidence from RCTs, our study provides evidence from rigorous causal analysis of high resolution retrospective data that mortality is decreased when a fluid cap of 6-10 L is maintained, with the greatest reduction at around 8L. This is broadly consistent with resuscitation volumes from the other retrospective studies above that show relatively lower mortality.

As in any observational study, there is no guarantee that we adjusted for all confounding variables. However, we believe that we adjusted for the most important drivers of treatment decisions pertaining to fluids. A helpful exercise is to compare the results of our analysis to what we would expect to see if we failed to adjust for important confounders. We would expect unobserved confounding to lead to monotonically decreasing estimated mortality rates as fluid volume caps decreased, since healthier patients tend to receive lower fluid volumes. Indeed, an *unadjusted* analysis estimates that 4 L fluid volume caps lead to a great reduction in mortality. However, Fig. 3 illustrates that our adjusted analysis estimated the highest mortality for the lowest fluid volume caps, which is an encouraging (though not definitive) sign that we successfully adjusted for confounding.

Other limitations to our study pertain to generalizability. First, this was a study of an ICU database at a single center and should be repeated with multicenter data. Second, to avoid bias, we had to exclude patients who violated any of our treatment strategies of interest (i.e., those who received over 4 L of fluid) before ICU admission. Our results are thus only applicable to the population of patients who arrive at the ICU without having already received large volumes of fluids. It is possible that effects of fluid caps would vary in the patients we omitted from our cohort. Third, we omitted patients with 0 L recorded pre-ICU fluid from our main analysis to guard against bias that might be induced by missing pre-ICU fluid data in this subpopulation. As a sensitivity analysis, we redid the analysis with these patients included and obtained qualitatively similar results (see Appendix E of Additional file 1). Fourth, we would ideally want to evaluate strategies governing treatment starting at sepsis onset, but because we only had detailed data beginning at ICU admission we focused on treatment decisions from that point onward. We mitigated this shortcoming by limiting our cohort to patients referred from the ED, which ensured that treatment had not begun too long before ICU admission for most patients in our sample. Finally, our data were collected from 2008 through 2012, and the effect of imposing fluid caps could have changed over time as fluid strategies have evolved.

We should also make the subtle point that our results are not necessarily estimates of the effects that would be observed if fluid caps were issued as guidelines. This is because we estimated the effect of abruptly cutting off fluids if physicians reached a (range of) threshold(s) after following usual care. If a guideline informed physicians of a fluid cap ahead of time, they may alter their treatment strategies in advance of reaching the cap in myriad ways (e.g., start vasopressors earlier, deliver lower volume boluses from time of admission, deliver less frequent boluses from time of admission, and administer maintenance fluid at a slower rate). If the distribution of treatment strategies in a world with a new guideline did not resemble the distribution of treatment strategies among patients whose care happened to be in accordance with that guideline in our data, then our results might not be good estimates of the outcomes that would be observed under the guideline. Thus, while our results are certainly evidence of benefit from fluid restrictive strategies, they do not point directly to specific guidelines.

## Conclusion

In this cohort study using causal inference methods in a large critical care database, we found that 30-day mortality would have likely decreased relative to observed mortality under current practice if these patients had been subject to “caps” on the total volume of fluid administered between 6 and 10 L, with the greatest mortality reduction at a cap of 8 L. Future multicenter retrospective studies, prospective studies, and RCTs are needed to further clarify the appropriate dose and timing of IV fluids in resuscitating septic patients.

## Supplementary information

**Additional file 1.** Supplementary materials.

## Data Availability

The data used for this study can be accessed via the MIMIC-III database (https://mimic.physionet.org/); full instructions for obtaining access can be found on the website.

## Abbreviations

Dyn-MSM: dynamic Marginal Structural Model
ED: Emergency department
EGDT: Early goal-directed therapy
ICU: Intensive care unit
IVF: Intravenous fluids
MIMIC-III: Medical Information Mart for Intensive Care III
RCT: Randomized controlled trial
SOFA: Sequential Organ Failure Assessment
