# Supplementary material for: Fluid-limiting treatment strategies among sepsis patients in the ICU: a retrospective causal analysis
Source: Crit Care. 2020 Feb 22;24:62. doi: 10.1186/s13054-020-2767-0 (PMC7036175; doi:10.1186/s13054-020-2767-0)

**Appendix A: Dynamic Marginal Structural Models (dyn-MSMs)**

See Orellana, Rotnitsky, and Robins (2009) or Hernan and Robins (2019) for a thorough review of dyn-MSMs for causal inference. Here, we provide a summary, linking them to our current application.

**Notation and Counterfactuals**

Let:

- $t\in\{ 0,\ldots,K\}$ denote hour after baseline, with K denoting end of followup, i.e. 24 hours;
- $A_{t}$be a variable denoting treatment at time t, i.e. fluids given;
- $L_{t}$ denote covariates at time t that may influence treatment decisions and be associated with the outcome, i.e. the confounders in our data;
- Y denote the outcome, i.e. 30 day mortality; and
- $\bar{X}_{t}$ denote patient history $X_{0},\ldots,X_{t}$ for arbitrary time varying variable X.

A treatment *strategy* is a set of rules or functions, one for each time point t that determine the value to which $A_{t}$will be set given observed history $(\bar{L}_{t},\bar{A}_{t-1})$. We denote arbitrary strategies by *g*, and we adopt the counterfactual framework of Robins (1986) in which Y(g) is a random variable representing the outcome that would be observed had strategy g been followed, possibly contrary to fact. Implicit in the notation Y(g) is the assumption that the treatment strategy followed by one patient does not influence the outcome of any other patient. This implicit assumption is called the `No Interference Assumption' by (Rubin, 1978). We make the additional assumptions:

**Positivity:** $P(A_{t}=a|\bar{L}_{t},\bar{A}_{t-1})>0$ for all a,t

**Consistency**: $Y=Y(g=\bar{A}_{K})$

**Sequential Exchangeability**: Y(g)$\coprod A_{t}|\bar{L}_{t},\bar{A}_{t-1}$ for all t, g

Positivity states that each patient has some chance of following each treatment strategy under consideration. Consistency is just a technical assumption necessary to link counterfactual to observed data. It says that the observed outcome is equal to the counterfactual outcome corresponding to the observed treatment. Sequential exchangeability basically means that there are no unobserved confounders of treatment at any time. It is satisfied, for example, if all causes of treatment that are also associated with the outcome are recorded.

A strategy is said to be static if its recommendation for the present does not depend on past covariate and treatment values. An example of a static strategy would be `give one liter of fluid every other hour'. A strategy is said to be dynamic if it does depend on past covariate and treatment values. An example of a dynamic strategy would be `give one liter of fluid if MAP<65 and total fluids administered so far <5L'. Our fluid cap strategies are dynamic as they depend on fluids given up to a given time. We are interested in estimating the population counterfactual mortality rate E[Y(g)] under strategies g that limit fluid volume.

**The dyn-MSM**

A dyn-MSM is a model for an expected counterfactual outcome under a class of strategies $\{g_{x}:x\in X\}$ parameterized by x as a function of x, i.e.

1. $E[Y\left( g_{x} \right)=f(x;\beta)$

In our application, x indicates fluid volume cap and *X* indicates the plausible range of caps we considered, i.e. 4L-12L. We specified the function *f()* to be a spline regression function. Under this model, if we knew $\beta$we could estimate the counterfactual mortality rate $E[Y(g_{x})]$ that would result from implementing fluid volume cap *x* for any *x.*

Estimation of $\beta$ proceeded in two steps.

**Step 1**: We separately estimated the effect $E[Y(g_{x})]$ of imposing each fluid cap x from a grid of candidate fluid cap values at 100 mL intervals from 4L to 12L. To estimate the population counterfactual 30 day mortality had a fluid cap been set at x mL:

· Step 1A: Fit a predictive model that outputs the probability of continuing to abide by the cap defined by x at hour t given that x was followed through hour t-1 and given covariate history through hour t. We used boosted trees for this step, specifically the R package xgboost. We set the maximum tree depth equal to 2, the learning rate equal to .3, and the number of iterations was chosen by cross validation.

· Step 1B: Weight each patient whose care actually follows cap x over the followup period by the inverse of the product of their conditional probabilities of continuing to follow x at each hour after baseline given their covariates up to that hour as estimated by the model fit in Step 1A.

· Step 1C: Compute the weighted average of the outcomes for patients who followed x using the weights computed in Step 1B. Assuming that the covariates used to fit the model in Step 1A satisfy the sequential exchangeability assumption, i.e. include all confounding variables, this is an estimate of the counterfactual average outcome had the guideline defined by X been imposed on the population.

**Step 2**: The estimates of counterfactual mortality under each x are somewhat noisy. We make the assumption that counterfactual mortality rates under volume caps vary smoothly with volume according to the dyn-MSM (1). We can estimate the parameters $\beta$ of this counterfactual regression by fitting a weighted regression to observed data. First note that each subject might follow multiple caps x. If a patient follows any cap, they also follow all greater caps. Let $\Lambda_{i}$ denote the number of strategies that subject *i* follows. Generate an artificial dataset with $\Lambda_{i}$ contributions from each subject: $(Y_{i},x_{i1},\ldots,\left( Y_{i},x_{i\Delta_{i}} \right).$ Using the artificial dataset, fit by weighted least squares the regression model E[Y|x] = f(x;$\lambda$) using the weights computed in Step 1B. If all confounding variables are included in the model from step 1A, the estimate $\hat{\lambda}$ of the weighted regression parameter approaches the causal estimand $\beta$.

We then repeat steps 1 and 2 on 500 bootstrap samples of the data to obtain confidence intervals for $\beta$and with it the estimated effect of each cap. We obtain simultaneous confidence intervals over effects of all caps using the method from Appendix C of van der Laan (2007).

**Appendix B: Feature Importance Table**

Table 3 ranks the contributions of each covariate to the probability of treatment model from Step 1B in Appendix A for x=4L. Gain is a measure of improvement in predictive performance resulting from inclusion of the feature. Frequency is a measure of how often trees in the ensemble chose to split on the variable. We can see that the variables that were important predictors of following the cap make sense and include most drivers of fluid administration decisions. This reassures us that our model is adjusting well for observed confounders.

| **Feature** | **Gain** | **Frequency** |
| --- | --- | --- |
| Total fluid volume by previous hour | 0.64 | 0.249 |
| Fluid volume administered in the previous hour | 0.079 | 0.067 |
| Running total urine output | 0.034 | 0.021 |
| Vasopressor rate | 0.026 | 0.036 |
| Systolic BP | 0.019 | 0.036 |
| Mechanical ventilation | 0.012 | 0.016 |
| MAP maximum | 0.012 | 0.01 |
| Pre-ICU fluid | 0.011 | 0.021 |
| Systolic BP maximum | 0.011 | 0.01 |
| Running total vasopressors given | 0.007 | 0.01 |
| Hour from baseline | 0.007 | 0.016 |
| Systolic BP minimum | 0.006 | 0.01 |
| Diastolic BP minimum | 0.005 | 0.005 |
| Cardiovascular SOFA minimum | 0.005 | 0.01 |
| MAP hourly difference minimum | 0.005 | 0.005 |
| MAP difference from previous hour | 0.005 | 0.021 |
| Weight (admission) | 0.005 | 0.016 |
| SpO2 minimum | 0.005 | 0.021 |
| GCS maximum | 0.004 | 0.005 |
| Running total vasopressors mean | 0.004 | 0.016 |
| Urine output mean | 0.004 | 0.01 |
| Heartrate minimum | 0.003 | 0.005 |
| GCS eyes component | 0.003 | 0.005 |
| Heartrate maximum | 0.003 | 0.01 |
| Cardiovascular SOFA mean | 0.003 | 0.01 |
| Last hourly MAP difference | 0.003 | 0.005 |
| Lactate minimum | 0.003 | 0.01 |
| Last systolic BP | 0.003 | 0.01 |
| Heartrate | 0.003 | 0.01 |
| Diastolic BP | 0.003 | 0.01 |
| Last urine output | 0.002 | 0.01 |
| Renal SOFA | 0.002 | 0.005 |
| Diastolic mean | 0.002 | 0.01 |
| Renal SOFA mean | 0.002 | 0.005 |
| Respiratory rate minimum | 0.002 | 0.016 |
| Vasopressor rate mean | 0.002 | 0.016 |
| Temperature maximum | 0.002 | 0.01 |
| Temperature minimum | 0.002 | 0.005 |
| GCS motor component | 0.002 | 0.01 |
| Last temperature | 0.002 | 0.01 |
| Cardiovascular SOFA | 0.002 | 0.005 |
| pCO2 | 0.002 | 0.005 |
| Hourly BP difference maximum | 0.002 | 0.01 |
| SpO2 max | 0.002 | 0.01 |
| last pCO2 | 0.002 | 0.01 |
| SpO2 mean | 0.001 | 0.005 |
| Urine output | 0.001 | 0.005 |
| GCS motor component minimum | 0.001 | 0.005 |
| CNS SOFA mean | 0.001 | 0.005 |
| CSRU | 0.001 | 0.005 |
| BUN max | 0.001 | 0.005 |
| CCU | 0.001 | 0.005 |
| Respiratory rate mean | 0.001 | 0.005 |
| Temperature mean | 0.001 | 0.005 |
| Respiratory rate maximum | 0.001 | 0.005 |
| Last lactate | 0.001 | 0.005 |
| SOFA mean | 0.001 | 0.005 |
| Liver SOFA minimum | 0.001 | 0.005 |
| Last MAP | 0.001 | 0.005 |
| Hourly BP difference mean | 0.001 | 0.005 |
| BUN mean | 0.001 | 0.005 |
| Oxygen therapy mean | 0.001 | 0.005 |
| Lactate max | 0.001 | 0.005 |
| MAP | 0.001 | 0.005 |
| BMI | 0.001 | 0.005 |
| Urine output minimum | 0.001 | 0.005 |
| Lactate mean | 0.001 | 0.005 |
| SICU | 0.001 | 0.005 |
| Platelet mean | 0.001 | 0.005 |
| Diuretic amount mean | 0.001 | 0.005 |

**Table 3. Feature Importance Table**. Means, minima, maxima, and totals are running tallies.

**Appendix C: R Code for dyn-MSM Analysis**

#Define treatment strategies in grid

min_start = 4000

max_start = 12000

grid = seq(from=min_start,to=max_start,by=100)

max_pre = min_start

#Load data (our main data frame is called hr_data)

setwd("~/projects/icu")

load("fluid_data_24hr.RData")

#Define which are treatment (A_vars), outcome (Y_vars), and confounding (L_vars) variables

A_vars = c(grep('^total_fluids',names(hr_data)),grep('^tev',names(hr_data)),grep('^amount',names(hr_data)))

A_var = which(names(hr_data)=='total_fluids')

Y_vars = grep('dod|Y_util|Y_surv|final_sofa|icu_los',names(hr_data))

L_vars = (1:ncol(hr_data))[-c(1,A_vars,Y_vars)]

#Load required packages

require(xgboost)

require(plyr)

require(rpart)

require(splines2)

require(glmnet)

#Force people to have at least some pre-icu fluid and be referred from the ED

hr_data = hr_data[hr_data$pre_icu_fluid>0 & hr_data$pre_icu_fluid<=max_pre,]

hr_data = hr_data[hr_data$icustay_id %in% ed_pats$icustay_id,]

hr_data = hr_data[order(hr_data$icustay_id,hr_data$hour),]

#Do 500 bootstrap runs

boot_runs=500

for(boot in 1:boot_runs){

#reset confounding variables at start of each run

L_vars = (1:ncol(hr_data))[-c(1,A_vars,Y_vars)]

#Make bootstrap sample dataset from original data

if(boot==1){

hr_data_boot = hr_data

}else{

samp_pats = sample(1:length(pats),length(pats),replace=T)

pat_inds = unlist(inds[samp_pats])

hr_data_boot = hr_data[pat_inds,]

hr_data_boot$icustay_id = factor(rep(1:length(samp_pats),lengths[samp_pats]))

}

#Fit predictive model for the outcome and adjust for predictions as confounders

X = model.matrix(as.formula(paste('Y_surv~',paste(names(hr_data)[L_vars],collapse = '+'),sep='')),model.frame(~.,hr_data_boot,na.action=na.pass))

niter_Y = 200

Y_mod = xgboost(verbose=0,data = X, label = hr_data_boot$Y_surv, max_depth = 2, eta=.3, nrounds = niter_Y, objective = "binary:logistic",missing=NA,metrics = 'rmse')

hr_data_boot$p_die = predict(Y_mod,newdata=X,missing=NA)

L_vars = unique(c(L_vars,which(names(hr_data_boot)=='p_die')))

imp_y = xgb.importance(Y_mod,feature_names = colnames(X))

#Keep track of outcomes, weights, etc for each treatment strategy

Y_list = vector(mode='list',length=length(grid))

weights_list = vector(mode='list',length=length(grid))

trunc_weights_list = vector(mode='list',length=length(grid))

x_list = vector(mode='list',length=length(grid))

pat_list = vector(mode='list',length=length(grid))

results = rep(NA,length(grid))

var_names_list = vector(mode='list',length=length(grid))

lambdas = rep(NA,length(grid))

data_boot_x_in_doubt_list = vector(mode='list',length=length(grid))

if(boot==1 & load_vars==F){

var_names = names(hr_data)[L_vars]

var_names = setdiff(names(hr_data)[L_vars],grep("meas",names(hr_data)[L_vars],value=T))

}

#For each treatment strategy, compute the weights for each patient who followed

for(j in 1:length(grid)){

x = grid[j]

data_boot_x = hr_data_boot

data_boot_x$followed = data_boot_x$total_fluids<=x

data_boot_x$disobeyed = unlist(aggregate(data_boot_x$followed,by=list(data_boot_x$icustay_id),function(x)cumsum(x==0)>0)[,2])

data_boot_x$past_disobeyed = unlist(aggregate(data_boot_x$disobeyed,by=list(data_boot_x$icustay_id),function(x)cumsum(cumsum(x))>1)[,2])

data_boot_x = data_boot_x[!data_boot_x$past_disobeyed,]

# print(table(data_boot_x$followed))

X = model.matrix(as.formula(paste('followed~',paste(intersect(var_names,names(hr_data_boot)),collapse = '+'),sep='')),model.frame(~.,data_boot_x,na.action=na.pass))

if(boot==1){

A_mod_cv = xgb.cv(verbose=0,data = X, label = data_boot_x[,'followed'], max_depth = 2, eta=.3, nrounds = 500,objective = "binary:logistic",metrics = 'rmse',missing=NA,nfold=10,early_stopping_rounds = 10)

niters[j] = A_mod_cv$best_iteration

}

fol_mod = xgboost(verbose=0,data = X, label = data_boot_x[,'followed'], max_depth = 2, eta=.3, nrounds = niters[j],objective = "binary:logistic",metrics = 'rmse',missing=NA)

data_boot_x$p_scores = predict(fol_mod,newdata=X,missing=NA)

cal_mod = isoreg(y=data_boot_x$followed,x=data_boot_x$p_scores)

yf = cal_mod$yf

yf[order(data_boot_x$p_scores)] = cal_mod$yf

data_boot_x$p_scores = yf

Y = unlist(aggregate(data_boot_x$dod,by=list(data_boot_x$icustay_id),function(x)ifelse(is.na(x[1]),0,x[1]/24 <= 30))[,2])

cens_pats = which(aggregate(data_boot_x$disobeyed,by=list(data_boot_x$icustay_id),function(x)sum(x)>0)[,2]==1)

if(boot==1){

pat_list[[j]] = pats[-cens_pats]

}

if(length(cens_pats)>0){

Y = Y[-cens_pats]

}

Y_list[[j]] = Y

weights = unlist(aggregate(data_boot_x$p_scores,by=list(data_boot_x$icustay_id),function(x) 1/prod(x))[,2])

if(length(cens_pats)>0){

weights = weights[-cens_pats]

}

weights_list[[j]] = weights

trunc_weights = pmin(weights,max(25,quantile(weights,.99)))

print(quantile(weights,.999))

trunc_weights_list[[j]] = trunc_weights

x_list[[j]] = rep(x,length(Y))

results[j] = sum(Y*trunc_weights)/sum(trunc_weights)

print(paste(boot,':',j))

}

#Record the results from the bootstrap sample

results_list[[boot]] = results

plot(grid,results)

outcomes[boot] = mean(hr_data_boot$Y_surv[hr_data_boot$time==1])

curve(0*x+outcomes[boot],from=min(grid),to=max(grid),col=4,add=T)

print(boot)

#Fit the dyn-MSM for the bootstrap sample using the computed weights

xs = unlist(x_list)

dyn_msm_mod_spline = lm(unlist(Y_list)~bSpline(unlist(x_list),knots=quantile(unlist(x_list),probs = c(.5))),weights=unlist(trunc_weights_list))

counterfactual_dih_probs_spline = predict(dyn_msm_mod_spline,newdata = data.frame(x_list=grid),type = 'response')

boot_results_spline[[boot]] = counterfactual_dih_probs_spline

points(grid,counterfactual_dih_probs_spline,type='l',col=2)

}

**Appendix D: Alternative Analysis Using Multiple Imputation and Logistic Regression**

As a sensitivity analysis, we estimated the parameters of the dyn-MSM described in Appendix A using an alternative method to estimate the inverse probability of treatment weights in Step 1B. In the analysis presented in the main body of the paper, we estimate these weights using boosted trees. There is no need to impute missing values as: (1) boosted trees accept NAs as inputs; and (2) NA is the appropriate value for a confounding variable that is unmeasured, since the doctor is also ignorant of its true value and it cannot influence treatment decisions. In this sensitivity analysis, we fit a logistic regression model to estimate probability of treatment. Since logistic regression does not accept missing values, we apply multiple imputation before fitting the logistic regression model.

More specifically, probability of treatment estimation in the sensitivity analysis proceeded as follows within each bootstrap sampled dataset:

Step 1: Generate 3 imputed datasets using the predictive mean matching method as implemented under the “pmm” option in the MICE package in R.

Step 2: For each cap, fit a pooled logistic regression model to each imputed dataset predicting whether the cap is exceeded at each time point. We included more covariates in models for caps that were more frequently exceeded. For caps that were exceeded by over 250 patients, we included as covariates in the logistic regression models: total fluid volume through the previous hour, fluid volume given during the previous hour, pre-icu fluid volume, total urine output, urine output during the previous hour, vasopressor rate, total vasopressors through the previous hour, systolic blood pressure, running minimum systolic blood pressure, diastolic blood pressure, running minimum diastolic blood pressure, systolic blood pressure in the previous hour, MAP, running minimum MAP, running maximum MAP, difference in MAP from previous to current hour, heartrate, running minimum heartrate, running maximum heartrate, respiratory rate, running mean respiratory rate, proportion of time on mechanical ventilation, hour after ICU admission, SOFA cardiovascular score, running mean SOFA cardiovascular score, weight,

pCO2, sPO2, GCS, GCS eyes component, GCS motor component, GCS running maximum,

lactate, lactate running minimum, SOFA renal score, SOFA CNS score, CSRU, CCU, SICU, temperature, temperature running maximum, temperature running minimum, probability of 30 day mortality from predictive model, and an interaction between fluid given the previous hour and change in MAP from the previous hour. For caps that were exceeded by over 100 patients but less than 250, we included as covariates: total fluid volume through the previous hour, fluid volume given during the previous hour, pre-icu fluid volume, total urine output, urine output during the previous hour, vasopressor rate, total vasopressors through the previous hour, MAP, running minimum MAP, running maximum MAP, difference in MAP from previous to current hour, heartrate, respiratory rate, proportion of time on mechanical ventilation, hour after ICU admission, SOFA cardiovascular score, weight, pCO2, sPO2, GCS, GCS eyes component, GCS motor component,lactate, SOFA renal score, SOFA CNS score, CSRU, CCU, SICU, temperature, and probability of 30 day mortality from predictive model. Finally, for caps that were exceeded by fewer than 100 patients we included: total fluid volume through the previous hour, fluid volume given during the previous hour, total urine output, vasopressor rate, MAP, proportion of time on mechanical ventilation, hour after ICU admission, weight, GCS, SOFA cardiovascular score, SOFA renal score, SOFA CNS score, CSRU, and temperature. Variables that were never equal to 0 were log transformed, and those that were sometimes zero were square root transformed.

Step 3: To estimate probability of exceeding a cap at a given timepoint, we averaged estimates from the three logistic regression models on the logit scale, then converted to a probability.

The figure below shows estimated effects of fluid caps between 4 and 12 liters with probability of treatment estimated as above. The results are very similar to **Figure 2** from the main body of the paper, indicating that results are not highly sensitive to the method used to estimate probability of treatment.

**Appendix E: Sensitivity to exclusion of patients with 0L pre-ICU fluid**

Many patients with 0L recorded pre-ICU fluids in reality received fluids that were not recorded. As total fluid is a very important variable (both as a confounder and as the basis for our treatment strategy definitions), including patients in whom this variable is missing could induce bias. This is why we excluded these patients from our main analysis. However, excluding these patients might negatively impact generalizability, so as a sensitivity analysis we reran our main analysis including patients with 0L recorded pre-ICU fluid. There were 3,326 patients in this larger cohort, and the mortality rate was 19% (compared to 17% in the cohort from the main analysis). Estimated effects of fluid caps were qualitatively similar to the main analysis.


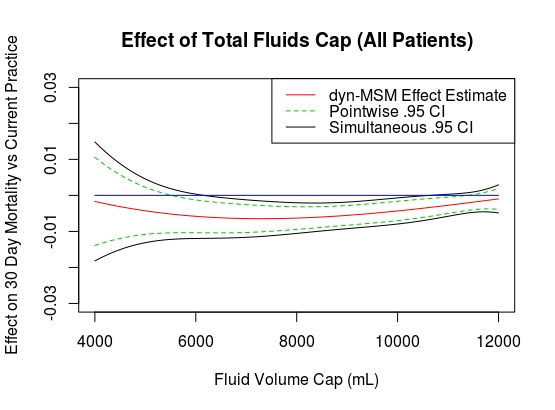

Supplement: Supplementary file 1 — Additional file 1. Supplementary materials. [file 13054_2020_2767_MOESM1_ESM.docx]
